# Transitions as experienced by persons in palliative care circumstances and their families – a qualitative meta-synthesis

**DOI:** 10.1186/s12904-018-0275-7

**Published:** 2018-02-05

**Authors:** André Fringer, Mareike Hechinger, Wilfried Schnepp

**Affiliations:** 1 Institute of Applied Nursing Science, University of Applied Sciences St. Gallen, Rosenbergstrasse 59, Postfach, 9001 St. Gallen, Switzerland; 2 Department of Nursing Science, Faculty of Health, Witten/Herdecke University, Stockumer Strasse 12, 58453 Witten, Germany

**Keywords:** Transitions, End of life, Palliative care, Informal caregiver, Family, Normality, Future, Hope

## Abstract

**Background:**

When receiving palliative care, patients and their families experience altered life situations in which they must negotiate challenges in daily life, increased care and new roles. With limited time, they also experience emotional changes that relate to their uncertain future. Transitions experienced in such situations are often studied by focusing on individual aspects, which are synthesized in the following study. The aim was to conduct a qualitative meta-synthesis to explore the experiences patients and their families gain during transitions in palliative care circumstances.

**Methods:**

A qualitative meta-synthesis was conducted following an inductive approach as proposed by Sandelowski and Barroso. Inclusion criteria were studies with adult persons in palliative situations and articles published in English or German. Relevant articles were identified by researching the Pubmed and Cinahl databases, as well as by hand searches in journals and reference lists for the period 2000–2015. The findings of each study were analyzed using initial coding, followed by axial and selective coding in this order. Consequently, a conceptual model was derived from the categories.

**Results:**

In total 2225 articles were identified in the literature search. Finally, 14 studies were included after the selection process. The central phenomenon observed among palliative care patients and their families was maintaining normality during transitions. Transitions are initially experienced unconsciously until a crisis occurs and responsive actions are necessary, which encourages patients and families to perceive the situation consciously and develop strategies for its negotiation. Patients remain caught between hopelessness and valuing their remaining time alive. As the illness progresses, informal caregivers reprioritize and balance their roles, and after death, family members inevitably find themselves in changed roles.

**Conclusions:**

In palliative care situations, transitions are experienced differently by patients and their families in a constant phenomenon that oscillates between unconscious and conscious perceptions of transitions. The derived conceptual model offers an additional perspective to existing models and helps to clarify the phenomenon in practical settings. The study promotes a differentiated conceptual view of transitions and emphasizes patients’ and families’ perspectives.

## Background

Transitions are a phenomenon of change affecting individuals and groups. In Transitions Theory, people are described as more vulnerable to health risks while experiencing transitions [1]. In palliative care, transitions are characterized by individuals experiencing a change due to deterioration or improvements of their health status. This can be changes of *place* (e.g. hospital to home, home to hospice), in *level of care* (e.g. informal care to professional health care, professional health care to professional *palliative* health care) or in *goals of care* (e.g. curative to palliative and no further treatment). Some transitions combine a change of place and level of care, e.g. persons with palliative care needs experience increasing care as their illness progresses and, when finally their needs cannot be fulfilled in the home, they are transferred to an inpatient hospice [2]. Individuals may experience multiple transitions during their illness [3]. Especially for individuals in a palliative context, the situation is characterized by various and continuous transitions that occur individually. For persons with palliative care needs, experiences of transitions are accompanied by distress and feelings of disruption [4], as transitions often occur suddenly and confront persons with palliative care needs and their families with new life situations. Family members and informal caregivers adopt new roles, accept responsibility and deal with own feelings while giving support to persons with palliative care needs during transitions [5, 6].

Research on this topic focuses on a) experiences of transitions in different patient groups, b) specific transitions like from curative to palliative, c) transitions in the health care system from one setting to another, or d) are related to transitions within a specific setting such as homecare or hospital [7–9]. A qualitative synthesis of existing findings to acquire an in-depth view on transitions experienced by persons with palliative care needs and their families, irrespective of their place of care or who cares for them, is lacking.

This paper intends to synthesize qualitative research about how transitions in palliative care circumstances are experienced by persons with palliative care needs due to cancerous diseases and their families. The European Association for Palliative Care (EAPC) [10] defines palliative care as an interdisciplinary approach for patients and their families to preserve quality of life. Care is provided through control of pain and other symptoms, as well as through support in social, psychological and spiritual problems. Relevant literature describes a controversial understanding of who is palliative and the conditions requiring palliative care (life-threatening, life-limiting, progressive (fatal) illness) [11]. The EAPC definition describing palliative care for patients “whose disease is not responsive to curative treatment” [10] is perceived as vague [11]. Therefore, the meta-synthesis defines persons receiving palliative care as those that are inducted into palliative treatment because of cancerous diseases and whose diseases will be fatal in the foreseeable future. The term *family* encompasses closely related persons and informal caregivers, regardless of a family relationship, and refers to those whom the patient includes in this group [12]. The following research question guided this study: How do persons in palliative care circumstances and their families experience transitions from receiving a palliative status until death?

## Methods

A mixed-methods studies review was originally planned to answer the research question. Therefore, the inclusion criteria were studies with adult persons in palliative situations published in English or German between 2000 and 2015. To include data for the focus of interest, no restrictions relating to study design or scientific merit were imposed [13, 14].

### Searching for and appraising the research reports

The literature search was performed between 06.10.2014–30.01.2015 (update 05.06.2015.) on the Pubmed and CINAHL databases. The search terms were: (Palliative care OR terminal care OR hospice care OR end of life) AND (patient OR informal caregiver OR next of kin OR family) AND (transition OR coping). Search terms were adjusted slightly to fit the different search systems, such as the use of MeSH terms in Pubmed. Additionally, hand searches in six palliative care journals (BMC Palliative Care, BMJ Supportive and Palliative Care, Journal of Hospice and Palliative Care Nursing, Journal of Palliative Care, Journal of Palliative Medicine, Palliative Medicine) and in reference lists of included studies were conducted. The literature search was set up broadly so that relevant studies could not be overlooked.

Two researchers independently screened the identified records for eligibility and discussed discrepancies. 31 studies suited the research topic. In a next step, two researchers read the studies meeting the inclusion criteria and appraised them separately using the checklists for qualitative research and cohort studies of the Critical Appraisal Skills Program (CASP) [15, 16] to systematically identify strengths and weaknesses of studies [17]. Results of the critical appraisal were compared and discrepancies discussed. Remaining discrepancies were discussed and clarified with the third researcher. The critical appraisal showed that the quality of studies identified was insufficient, especially the quantitative ones. Therefore, the available qualitative studies that received a good appraisal were used for a qualitative meta-synthesis. Qualitative studies with slightly lower quality were used if their content held relevant information. A meta-synthesis is a method to obtain an interpretive integration of qualitative results offering a fully integrated description of the research issue instead of a summary of unlinked features. The meta-synthesis followed the recommended steps of Sandelowski and Barroso’s (2007): formulating a purpose (1), searching for and retrieving qualitative research reports (2), appraising the research reports (3), classifying the findings (4), conducting a meta-summary (5) and developing a meta-synthesis (6) [14].

### Classifying the findings, conducting a meta-summary and developing a meta-synthesis

In a qualitative meta-synthesis, findings of studies should be read in regard to what they reveal about the specific measures that were applied. Therefore, it is important to be aware of how the findings were interpreted. Studies included in the meta-synthesis as recommended were classified with a typology [14]. The included studies were located in one of the following classifications: thematic surveys, conceptual/ thematic descriptions, or interpretive explanations.

The basic assumption for conducting a meta-summary and developing a meta-synthesis was that the findings of the included studies are the researchers’ interpretations of their collected data. Consequently, the written findings of the included studies were treated like a transcript in a qualitative study. Therefore, the results section of each study was used as data for the meta-synthesis. Analysis started by re-reading the studies several times in a process of familiarization. Initial coding as an inductive analysis procedure was used as the first cycle method, followed by axial and selective coding as second and third cycle method in this order [18]. The aim was to reflect deeply on the contents of the data and to gain higher levels of abstraction and conceptualization with ongoing coding. This method of constant comparison enabled the identification of similarities and differences. As coding is an iterative and cycling process, codes were re-examined by three researchers several times. Core components of the inductive meta-summary were initial and axial coding showing a quantitatively oriented aggregation of the findings. The codes and categories were then synthesized through further analysis in axial and selective coding [18]. Finally, a conceptual model was derived on the main categories. The qualitative data analysis software MAXqda12 supported data analysis process.

Furthermore, results were counterchecked with the article by Penrod et al. [19], which is a grounded theory of family caregivers’ experiences in end-of-life caregiving. Penrod et al. [19] derived from their analysis a model of caregiving through the end of life. The model comprises experiences and key transitions of informal caregivers from prediagnosis through the end of life. The main process describes informal caregivers “seeking normal”. Since Penrod’s article contains a comprehensive grounded middle-range theory with strong emphasis on illness trajectories, the results were not included to ensure the inductive analysis process.

## Results

**Fig. 1:**
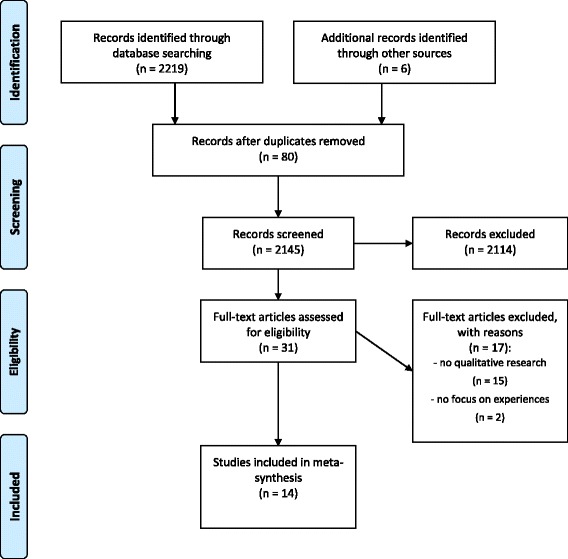
Flow chart on study selection process according to the PRISMA guideline [43]

In total, the literature search yielded 2219 records from databases and 6 from the hand search. The records were imported into a reference management system. 2145 records remained after deletion of the duplicates. Two researchers independently screened the title and abstract for eligibility and discussed discrepancies. In a next step, two researchers read the identified 31 full-texts and used the Critical Appraisal Skills Program. A qualitative meta-synthesis was conducted instead of a mixed studies review, due to the poor quality of the identified quantitative studies. Fourteen studies were included in the meta-synthesis after discussion of eligibility and critical appraisal within the research team. Fig. 1 presents the flow of articles included and excluded.

Characteristics of the included articles like country, setting and sample, focus of interest, applied method and analysis were extracted and summarized in Table 1. The included studies refer to persons with palliative care needs and their families. The studies use the terms “informal caregivers” or “family members” to describe families. The terms are used as referenced in the studies, depending on whether the included studies focus on individuals caring for the persons with palliative care needs or on family members.

**Table 1:** Characteristics of studies included in the meta-synthesis

*Author (year)*	*Country of study*	*Setting/ Sample*	*Focus of interest*	*Method/ Analysis*
*Berterö* et al. *(2008)*	Sweden	23 persons with palliative care needs	Persons with palliative care needs’ experiences of receiving a diagnosis of an inoperable lung cancer and its impact on their life situation and quality of life	Interviews/ interpretive phenomenology
*Brajtman (2003)*	Israel	26 family members 6 hospice staff	Family members’ experiences, needs and feelings of terminal restlessness	Focus groups and interviews/ Phenomenological approach
*Carlander* et al. *(2011)*	Sweden	10 informal caregivers	Informal caregivers’ perceptions of situations in daily life that are challenging their self-image when caring for a person with palliative care needs	Interview/ interpretive description approach
*Clemmer* et al. *(2008)*	Canada	4 informal caregivers	Family members’ experiences of roles while providing home-based palliative care	Secondary analysis of in-depth interviews/ ethnographic approach
*Duggleby* et al. *(2010)*	Canada	6 persons with palliative care needs, 10 informal caregivers, 12 health care professionals	Persons with palliative care needs’ and informal caregivers’ experiences of transitions when receiving palliative home care	Focus groups and open-ended interviews/ Grounded theory approach
*Groot* et al. *(2007)*	Netherlands	1 person with palliative care needs and 1 family member	Experiences of a married couple in a palliative situation	In-depth interview / Systematic content analysis
*Harding & Higginson (2001)*	England (UK)	14 current and 4 bereaved informal caregivers	Informal caregivers’ perceptions of obstacles in the process of accessing appropriate support and during provision of targeted interventions	Semi-structured interviews/ analytical methods of Grounded Theory
*Hebert* et al. *(2009)*	USA	33 informal caregivers of terminally ill patients	Informal caregivers’ perceptions of factors that they believe are important to prepare for death and bereavement	Focus groups and ethnographic interviews/ constant comparative method
*Holtslander* et al. *(2005)*	Canada	10 informal caregivers	Informal caregivers’ experiences of hope	Open-ended interviews/ Grounded Theory approach
*Olsson* et al. *(2011)*	Sweden	11 persons with palliative care needs	Persons with palliative care needs’ experiences of hope	Interviews, diaries and questionnaires/ Grounded Theory approach
*Sand* et al. *(2009)*	Sweden	20 persons with palliative care needs	Persons with palliative care needs’ coping strategies in the presence of their own impending death	In-depth interviews/ hermeneutic interpretative method
*Steinvall* et al. *(2011)*	Sweden	11 family members	Family members’ experiences of quality of life and their life situation with persons with palliative care needs	Interviews/ phenomenological approach
*Sutherland (2009)*	Canada	8 female family members	Family members’ perceptions of transitions at the end of life	Interviews/ phenomenological approach
*Syrén* et al. *(2006)*	Sweden	5 families	Families’ perceptions of being a family when one family member is terminally ill	Narrative interviews/ phenomenological approach

**Fig. 2:**
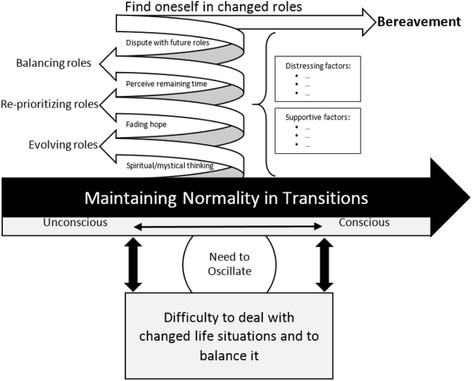
Conceptual Model: Transitions experienced by persons in palliative care circumstances and their families

Based on the meta-synthesis of the qualitative findings a conceptual model of transitions experienced by persons in palliative care circumstances and their families was derived (see Fig. 2) as a central result of this study. The main category of concerned persons and their families is “Maintaining normality in transition” which contains the following subcategories: distressing and supporting factors influence them in adapting to the situation (1). While experiencing transitions they have to find themselves in changed roles (2). Furthermore, they anticipate the future after the person with palliative care needs has died (3). In the following, the main category with the subcategories will be presented.

### Maintaining normality in transitions

The diagnosis of an incurable oncologic disease is a devastating experience for persons with palliative care needs and their families. They experience difficulties when dealing with changed life situations. They try to adapt to this unprecedented situation by maintaining “normality” and routines in daily life as far as possible, despite facing the prospect of death. Persons with palliative care needs and their families oscillate in an attempt to find balance in the changing circumstances of everyday life. The process of adapting to transition is described as “Navigating Unknown Waters” [4].

#### Adapting to a life with an incurable disease by redefining “normality”

When receiving the diagnosis of an incurable oncologic disease it is important for persons with palliative care needs to receive support from their families. They wish to talk about their feelings, their altered life situation and their worries [20]. Persons with palliative care needs and family members often experience uncertainty and fear in this phase, as they are confronted with limited time and feelings of “disruption” that diminish quality of life [20, 21]. They know that the “Sword of Damocles” is hanging over them [22]. In order to counterbalance these feelings, a “process of redefining normality” begins [4]. After enduring the situation passively, persons with palliative care needs and their families start to take measures, depending on individual support and personality. Persons with palliative care needs state that they are “not ready to leave life” as “there (are) a lot of things left to do” [20].

#### The patient’s perspective

Persons with palliative care needs develop several strategies to achieve *normality* in everyday life with their disease. They want to be treated as the same persons they were before the diagnosis of an incurable oncologic disease and hence maintain their independency and integrity. Further, they like to be involved in daily life with family and friends [20, 23]. Thinking of death provokes painful feelings, and persons with palliative care needs actively try to restrain these feelings within endurable limits so that they do not “consume their whole existence” [23]. They strive to maintain daily routines and to preserve a balance in their lives [20]. This results in a focus on the especially precious “borrowed time”, which leads persons with palliative care needs to define new priorities and preferences for the limited time they have at their disposal [22]. They actively decide how to pass this time “to create a bearable existence in the face of death “ [23].

Daily routine provides a distraction from the illness and has a relieving effect on persons with palliative care needs. However, participating in everyday life depends on the intensity of symptoms [20, 23]. Persons with palliative care needs try to actively control their symptoms [20], which helps them “to keep death at a discreet distance” for as long as possible [23]. Persons with palliative care needs experience a “still functioning” body as reassuring and as a “confirmation” of life. In contrast, confrontation with certain symptoms is an alarming experience. Nights are particularly distressing, as they are exposed to the “ordeal” of an uncontrollable stream of thoughts [23]. Symptoms are described as sometimes worse than the disease itself [20]. Persons with palliative care needs can acknowledge the disease and the changed life situation through reminiscing and reframing hope [4].

#### The family perspective

Family members and informal caregivers live through the same process of redefining normality as persons with palliative care needs. Informal caregivers develop a modified self as they have to stretch limits, as well as challenge ideals and interdependencies [24]. Family members and informal caregivers maintain daily life through planning in the short term, but always with more or less explicit contingencies, as daily planning depends on the ill person’s health status [22, 25, 26]. Holtslander et al. [27] identify four sub-processes that can be seen in the context of daily routine: “doing what you have to do”, “living in the moment”, “staying positive” and “writing your own story”. There is an upward trend in these processes that begins with “doing what you have to do” that allows the informal caregivers to progress to the other processes.

Family members experience difficulties to "achieve a balance […] to deal with their own lives and (with) their everyday life with the ill person, balancing between the old and the new life situations” [26]. They are torn between satisfying their own needs and *not* engaging with their own needs and anxieties [26, 28]. They cling to everyday life and avoid issues like sorrow and emotional distress [20]. To maintain everyday life, informal caregivers have to “recharge their batteries” on a regular basis and do something for themselves [27]. Possibilities to recuperate are going for a walk, working, leaving for a weekend trip or taking some time to pray or read [24, 27]. Informal caregivers describe the presence of a nurse or doctor with the persons with palliative care needs as “safe time” to organize something or “build up physical strength to care” [28]. Although time-outs from their caring duty are desired by family members and informal caregivers, they feel reluctant to leave the person in the care of somebody else [28] as they want to make use of the remaining time with the loved one [25]. They appreciate the time together and “take each day as it comes, do their best and a little extra” [26].

#### Social environment of transitioning

Persons with palliative care needs and family members/informal caregivers encounter transitions as individuals *and* together as a family. They adapt their life situations to these transitions. Generally, adaptation can be divided into unconscious (passive) and conscious (active) forms. Unconscious adaptations occur constantly and are characterized by passive responses on the part of the individual and by letting things happen. Syrén et al. state that this behavior is combined with insecurity, withdrawal and a sense of helplessness. Helplessness is compounded by a lack of resources inside and outside the family [21]. As an example, increasing dependency on care in the palliative situation is perceived initially as an unconscious process until finally the situation cannot be dealt with anymore. This marks the transition that also can be seen as a crisis of understanding the crisis as a situation in which changes in behavior, reorientations and decisions become necessary. The transition is noticed consciously and persons with palliative care needs, as well as their families, actively elaborate new strategies that enable the maintenance of normality. For Syrén et al. [21], an active behavior comes about through openness and comfort, e.g. sharing thoughts with each other, togetherness and the will of family members to prevent concerned persons from suffering. The availability of resources inside and outside the family helps to remain in control of the situation [21]. Inner strength is gained and defined through hope [27] that is seen as “a companion, a fellow traveler “and can be influenced from the outside, e.g. the family [29]. Therefore, families can increase the quality of life of persons with palliative care needs [20]. Reciprocity is an important resource, as the network of friends and community provide emotional and practical support that helps to manage transitions [4, 20, 27]. Persons with palliative care needs and their families adapt by maintaining normality. Daily routines are preserved until another phenomenon occurs (e.g. new or worsened symptoms) and again, unconscious and later conscious adaptations are made. Therefore, concerned persons and their families vacillate between conscious and unconscious perceptions of transitions. Supportive and distressing factors can also affect the experience of transitions (Table 2).

**Table 2:** Identified supportive and distressing factors

Supporting factors
• Experiencing continuity and stability as a precondition for maintaining a balance while “navigating unknown waters” [4].
• Adopting an active attitude towards challenging and continuously changing situations [4, 22]
• Being able to redefine normality in one’s life situation, thereby creating new possibilities of well-being in the context of an incurable illness [4].
• Acknowledging the present situation and being satisfied with it [22, 27]
• Being able to keep hope alive [27, 29]
• Experiencing family cohesion and support [21, 23, 26]
• Using openness and honesty in communication to handle the situation and support each other [21, 26, 30]
• Relying on positive memories of the past as a basis for accepting the present situation [4, 23]
• Receiving support by professionals [20, 25, 28–30]
• Using magical thinking and memories of the past [23]
Distressing factors
• Experiencing disruption and instability [21] as a reason of losing one’s balance while “navigating unknown waters” [4]
• Not being able to adopt an active attitude [22].
• Not being able to redefine one’s life situation in the context of an incurable illness [4].
• Being exposed to distress, anxiety and uncertainty leads to a feeling of losing control because of not knowing what to expect [4, 21].
• Vulnerability of the sick persons influence the family members’ lives and was on their mind [22]
• Experiencing fears about the future [22].
• Losing hope [27, 29]
• Experiencing a lack of family cohesion and support [21]
• Missing authentic communication [21, 26]
• Missing support by professionals and experiencing difficulties with the health care system [25, 31]

Persons with palliative care needs and their family members manage the palliative situation and their transitions differently, depending on the use of resources in the family. The main aspect is to be aware of the continuous passive changes and to become an active creator of transition. Encountering a crisis cannot be avoided as the illness progresses and transitions occur; but persons with palliative care needs and family members can still adjust their daily life actively to maintain normality.

### Experiencing transitions and finding oneself in changed roles

While facing transitions and dealing with a life-limiting illness persons with palliative care needs and their families are confronted with new challenges. Persons with palliative care needs experience difficulties in finding their purpose in life and coping with their role as patients being faced with increasing dependency from care. Meanwhile family members experience an adjustment in their roles.

#### Burdens and fears in the context of transitions

Family members and persons with palliative care needs experience the strains associated with the palliative process mainly as tentativeness, anxiety and distress [20, 21, 23, 25]. Uncertainty occurs due to the restrictions on making plans for the future due to disease progression [25], as well as in regard to whether there will be enough time “to conclude their lives” [20]. Family members report feelings of helplessness when they do not have sufficient resources to understand and manage a situation caused by the disease [21]. Feelings of separation, isolation, powerlessness and forlornness emerge as the disease progresses [23, 25]. Some persons with palliative care needs try to deal with their fear by talking about it. They speak with family members about death, while others do not want to talk about dying as they think there is enough time left [20]. Often family members grieve about the progression of illness as they are left watching and waiting for its progression [30]. Although activities and distractions help informal caregivers as forms of coping, they experience distress because of concerns for the persons with palliative care needs [28]. Moreover, informal caregivers describe losing their identity with ongoing care because they feel either "invisible or are just a part of the patient" [28]. Therefore, informal caregivers encounter difficulties separating the ill person’s experience from their own [24], e.g. when they are asked how they feel they answer by describing the condition of the person they care for.

#### Changing relationships between persons with palliative care needs and their families and friends

In a palliative context, the relationships between persons with palliative care needs and their partners, family members or informal caregivers often become closer and more intimate [4, 24, 27, 30]. Some family members review their past relationship with the persons with palliative care needs, which can lead to satisfaction or questioning the development. Other family members seek meaning in their relationship and “acknowledge their imperfect lives” [30]. Talking with each other helps informal caregivers to acquire a better understanding of each other’s hopes, feelings and frustrations [27]. Informal caregivers describe the relationship to close persons with palliative care needs as a “greater appreciation for their own lives and health, including feelings of being more vivid and an enhanced sense of life” [24].

#### Hope as a meaningful phenomenon

Hope is a constant phenomenon that accompanies persons with palliative care needs and family members when experiencing a life-limiting illness. Olsson et al. describe hope as glimmering embers glowing with different strengths, from a glimmer to a spark to a flame – changing from one moment to the next [29]. This relates to the varying intensity of hope in different situations, which is influenced in turn by current health status, well-being or information levels of the persons with palliative care needs. As the flame only needs a tiny spark, persons with palliative care needs only require a tiny amount of information to develop hope. The embers represent the core process from which four subprocesses emerge. The identified processes may change order or repeat themselves [29]. Hope is fluctuating and not steady and may even change over the course of the day [27, 29]. Hope leads to a feeling of freedom and independence for persons with palliative care needs and provides the persons with palliative care needs with meaning in life [29], while hope is tantamount to inner strength and a source of courage for informal caregivers [27].

The first process – “convinced hope” – characterizes the persons with palliative care needs’ urge to look forward [29]. Persons with palliative care needs wish for a future without distressing symptoms, that they can stay at home or do not become lonely [23]. They experience well-being, strength, motivation and courage to keep fighting [22, 29]. Persons with palliative care needs, family members and informal caregivers value hope immensely as it helps them to cope with difficult situations and to maintain a positive attitude, as well as preventing them from being overwhelmed by negative feelings associated with death and finiteness [20, 23, 27]. Persons with palliative care needs try to convince themselves and others of their hope [29]. If the persons with palliative care needs keeps hope alive – even if there are no prospects for recovery − this has an encouraging effect on the informal caregivers as well [27]. The strong feeling of hope does not permit a reflection on death for persons with palliative care needs, so “their own death was still a far-off concept rather than a reality. Yet they were in a palliative context” [29].

The second subprocess – “simulated hope” – relates to unrealistic thinking [29]. Persons with palliative care needs often cling to hope instead of confronting the facts. Believing in miracles is a widespread phenomenon among persons with palliative care needs [22, 29] and informal caregivers [27]. They describe having “the courage to hope but thought that it might not be correct or safe to truly believe in it” [29]. Keeping hope alive is also essential for family members. They experience an increasing awareness about the imminent death of persons with palliative care needs, while at the same time they rely on hope for recovery. Sometimes even a small piece of information can inspire a ray of hope. Encouraging statements by physicians and “good news” about laboratory results serve as external sources of hope [26]. Statements by health professionals, especially nurses, have a high influence on the intensity of hope experienced by persons with palliative care needs [20]. Persons with palliative care needs think it is better to “hold on to a grain of hope than to have no hope at all” [29]. Persons with palliative care needs cling to hope, even if there are no prospects of improvement [30].

"Collecting and maintaining moments of hope” − the third subprocess – describes an awareness of the palliative situation and living for the moment [29]. Persons with palliative care needs acknowledge the life-limiting illness and how little they can influence the situation, but still want to seize the day [29] as they are “not even close to denial” [23]. They want to enjoy the limited time and hope for a good quality of life [20]. Informal caregivers describe “living in the moment” as a strategy to focus on the actual moment and not to become overwhelmed by thoughts about the future [27]. Therefore, family members and persons with palliative care needs focus on living from day to day and appreciate the remaining time they can share [25]. They spend time consciously, collect meaningful moments and distract themselves through focusing on pleasant experiences [26, 29]. Persons with palliative care needs perceive the present in an intensified way [23].

The fourth subprocess − “gradually extinguishing hope” − is associated with a recognition by the persons with palliative care needs that their time is coming to an end [29]. Hope proves to be a synonym for the will to live. Some persons with palliative care needs fear that losing hope will lead to imminent death. Hope fades when they lose their meaning for staying alive. Signs of the progressing illness, bad news or the awareness that they are no longer able to influence the situation can abruptly diminish hope. A letting go of energy accompanies this fading hope. It is a slow fading of hope. They want to think about the future, but have lost the courage to do so. These persons with palliative care needs often “felt they were losing all hope and would be forced to literally spend the rest of their lives waiting to die” while others “completely lost all their strength and the ability to care about anything that happened around them or to themselves." [29]. With “eroding hope” family members’ hope is described as beginning to weaken due to several factors, e.g. the deteriorating health status of the persons with palliative care needs, the receipt of negative news concerning the condition of the persons with palliative care needs or unfavorable experiences with health care professionals. But some hope will always remain nonetheless [27].

#### Magical thinking and imagination

Many persons with palliative care needs use memories of the past, magical thinking and imagination, e.g. daydreams and fantasies, to counterbalance negative experiences. They seek possibilities to escape from reality into a “make-believe world” for a few moments. Some persons with palliative care needs develop deep empathy with other persons, nature and animals. Perceiving togetherness with nature offers a sense of belonging to a cohesive whole and of feeling connected to “a higher power, a spiritual guide, a watching hand or just a greater something” [23]. Informal caregivers also nurture hope by connecting to a spiritual aspect, with something greater than oneself [27]. This kind of spiritual development is comprehensible in the context of a life-limiting disease. There is hope for every opportunity to acquire additional time alive, and persons with palliative care needs use courage and hope as a means of fighting their illness. Some turn to alternative and homoeopathic treatment methods [29]. Persons with palliative care needs want to do something, even if its efficacy is obscure [4, 22]. This tentative process is described as “Counterbalancing Death with Manifestations of Life” [23]. However, turning to spiritual ways of thinking and alternative medicine does not exclude confronting oneself with reality and facing symptoms with “strength, courage, perseverance, a sense of humor and a capacity not to get too engrossed in dark thoughts” [23].

#### Protecting one another

During transitions, mutual protection is an identifiable phenomenon that plays out between persons with palliative care needs and family members. Persons with palliative care needs want to protect their family and friends from the implications of their disease [20], while family members seek to protect persons with palliative care needs from unpleasant feelings [26]. Therefore, family members often do not show sadness and are more prone to telling persons with palliative care needs positive things, masking their own strain and stress [26]. For persons with palliative care needs, it was difficult to admit having a serious illness to themselves and consequently to share details with family and friends [20]. As persons with palliative care needs do not want to reveal details of their disease, it becomes difficult for them to behave authentically. Some even try to “downplay” their situation by changing the vocabulary, for example by avoiding the term “tumor” and using words like “dots”, “bubble”, “him” or “it” instead [23]. Moreover, truthfulness with friends changes also, as persons with palliative care needs do not pass on bad news and instead try to appear confident and courageous [20]. In consequence, family members and persons with palliative care needs find themselves in an ambivalent situation between protecting each other and behaving authentically. This is shown in family members wanting to support persons with palliative care needs and to provide a sense of security, while at the same time trying to feel comfortable themselves and remain in control of the situation [26]. As the illness progresses, protecting family members becomes a balancing act, as persons with palliative care needs want to support family members so that they can manage the time afterwards as well [20]. Moreover, the progressive illness decreases the ability of persons with palliative care needs to care for themselves, and being dependent on support from others is a frustrating experience for persons with palliative care needs [4]. It is a decisive event when the condition of the persons with palliative care needs deteriorates, symptoms increase and it is no longer possible for them to fulfil professional and family tasks, resulting in an adjustment in routine daily activities [4, 23]. Persons with palliative care needs feel “exposed to the vagary of existence” [23].

#### Family members and informal caregivers adjust to new roles

The adjustment of roles in transitions is identified in a dynamic process that involves three intertwined themes (Table 3) [25]. Family members experience *evolving roles* in the process of accompanying the requirements of a person with palliative care needs, which produces new tasks. As time passes, *re-prioritizing* of roles is necessary, as family members have to decide which role is given precedence, e.g. caring, earning money, being a mother, sister or wife. Consequently, roles have to be *balanced*. Multiple demands on one specific role could lead to over-exertion, and family members must find balance in their different roles [25].

**Table 3:** Family members’ and informal caregivers’ role adjustment

	Family members/ Informal caregivers…
Evolving roles	• Have to evaluate their needs and decide referring to multiple expectations whether to assume the caregiver’s role or not. The decision depends on social claim, life roles and gender expectations [24, 25] as well as spiritual, social and moral obligations [32].
	• Experience changing roles inside the family and take over multiple roles [32].
	• Have to meet expectations which is a central challenge [25].
	• Are confronted with new tasks and find themselves in a changed life situation [26].
	• Have to deal with an increasing workload because of previous tasks extended through new tasks like accompanying, supporting and caring for persons with palliative care needs [26] as well as to coordinate with health care professionals [25].
	• Have to negotiate boundaries between family and professional caregivers [25].
Re-prioritizing roles	• Have little scope in decision-making as it is expected to prioritize the caring role more than e.g. the employee role [25].
	• Missing other options as the willingness to care diminishes other opportunities [28].
	• Care as commitment for their family members [32].
	• State that it is self-evident and natural to care for a family member [24].
	• Re-prioritize values and expectations in the family [25].
	• Do not watch their own health out of a sense of duty wherefore own needs are postponed and the concerned persons’ needs are put before their own [24, 28, 32].
	• Describe the phenomenon as: “We just do what we have to do, and get through it”; which is combined with strategies as to accept the situation and not to give up [27].
Balancing roles	• Hope that “they would be able to handle whatever the future would bring and to do a ‘good job’ of caregiving without giving up […]” [27].
	• Experience emotions as pendulum-like, a reciprocity between the need to care and to fulfil expectations at the same moment from persons with palliative care needs, health care professionals, society and their own.
	• Try to “hang on” to provided expectations and deal with the increasing interdependency [24, 25, 28].

### Anticipating the future

An important aspect of transitions in the palliative context is related to anticipations of the future for persons with palliative care needs and their families. Nevertheless, they try to be prepared and to deal with life in the shadow of death.

#### Confrontation with an unknown future

Imagining how family members will live after the death of the persons with palliative care needs proves meaningful for persons with palliative care needs and family members alike [22]. Persons with palliative care needs think about the future by referring to what family members will miss about them [30]. Persons with palliative care needs experience it as emotionally distressing to think about how daily life goes on without them. Their worries concern the response of children and parents and how they will manage the situation after death. Some persons with palliative care needs ask themselves how their children will react to their death and how they will go on living without a father or a mother. It is very important for persons with palliative care needs to be certain that their children receive support [20]. It is essential to them to remain “present” for posterity, even after their death. For example, the skills they taught their children or shared memories should be helpful for family members in their future life [23]. However, persons with palliative care needs are painfully aware of the fact that they will never witness future life events like the birth of their daughters’ babies or their spouses’ retirement from work [22]. Some persons with palliative care needs are sadder to leave their loved ones than they are afraid of death [20]. For others it is frightening to imagine that there is no life after death, and so they feel comfort to “hold on to the possibility of a something even if it was nothing tangible” [23].

For caring relatives, it proves very difficult to face up to a future without the deceased relative. Relatives are concerned about living without their marriage partner out of personal, practical and financial considerations. Managing the future alone is a great worry. Even while the persons with palliative care needs are still alive, they have a fearful presentiment of being lonely in the future and reflect on the consequences [26, 30]. However, family caregivers try to “prepare” themselves for the imminent death of the persons with palliative care needs.

#### Being prepared

“Preparing” oneself for loss is a significant issue for family members and informal caregivers in the context of transitions. Hebert et al. state that life experience and a long caring relationship is not sufficient to prepare for care, death and bereavement [31]. Although the awareness of the disease and its consequences exist, family members have difficulty preparing themselves, which leads to uncertainty and anxiety [26].

In the context of preparing, Hebert et al. identify three dimensions. The “cognitive dimension” is related to informal caregivers receiving “medical, practical, psychosocial, or religious/spiritual information” [31]. The second dimension is associated with preparing “mentally” or “emotionally” in order to bid farewell to persons with palliative care needs [32] and to conclude this period of life at peace [31]. In respect to the time after death, family members prepare themselves for the loss of roles before the situation arises [25]. The third dimension is concerned with behavioral strategies, e.g. organizing practical matters or financial affairs. Every family member or informal caregiver uses different strategies, depending on the duration of illness, prior instructions with regard to death, as well as previous experiences with caring for a dying person [31]. “Preparing” starts early − even at the time when an incurable oncologic disease is diagnosed [26]. Some family members or informal caregivers soon become aware of the necessity to confront the approaching loss, as well as their anxieties concerning their personal future after the death of the persons with palliative care needs [28]. This “preparatory” work proves essential for accompanying the dying persons with palliative care needs, as well as for overcoming the transition associated with loss and bereavement [31].

#### Accompanying the dying patient

The time immediately before the death of the persons with palliative care needs death is extremely demanding and intense for family caregivers. The role of the caregivers expands in a proportionate relationship to the deterioration in health status of the persons with palliative care needs [25]. Caregiving receives the highest priority and attention during the final weeks, days and hours in the lives of the persons with palliative care needs. Informal caregivers avoid leaving the persons with palliative care needs alone at home. The significant vulnerability of the persons with palliative care needs is constantly on their minds [22]. The physical and emotional strain on family members and informal caregivers increases as a result. They dedicate all their energy to the persons with palliative care needs − at the expense of their personal needs and their own health. In general, they consider their personal health less important compared to the critical situation of the persons with palliative care needs between life and death [25].

To provide support for a dying relative, caregivers have “to stretch their limits of intimacy and privacy “[24]. Family members describe the dying persons’ suffering as physical, emotional and/or spiritual distress. Particularly prominent for them are the strong emotions of the dying relative, expressed in a “dramatic physical manner, such as feelings of loss of control, anger, frustration and fear “[32]. Nevertheless, family members try to understand the different emotions of their partners [30]. For some caregivers, “dying and death may be so hard to bear and to think of that families are not able to find peace in the present at all. Death is expressed as disgusting and as a termination which is almost impossible to consider” [21]. Informal caregivers are sometimes desperate and admit to drinking alcohol to numb their thoughts or using earplugs to avoid hearing the rattle of breathing. Other thoughts are directed at violence toward other family members or shortening the care recipient’s life. These thoughts and feelings are characterized as forbidden. Sometimes they are unable to recognize themselves based on their responses in different situations [24].

Ensuring that the dying person experiences only minimal pain and suffering is a major concern for informal caregivers [27]. In this context, the question of sedative medication arises. Family members have an ambivalent attitude toward sedation. On the one hand, they want “the patient’s suffering to end” [32]. On the other hand, sedation results in “reducing, altering, or effectively ending their communication” with the dying persons with palliative care needs [32]. This contradicts the family member’s wish to take over emotional and physical burdens from the dying persons with palliative care needs in order to relieve them and let them die peacefully [30]. As this is not possible if the dying persons with palliative care needs is sedated, family members express “sadness and regret over their inability to communicate with their relatives before their death” [32]. As death approaches, the need to talk to the dying relative gains increasing significance for family members [32]. In the face of death, an informal caregiver stated that “it felt good to be together and to share thoughts and feelings with the dying loved one” [24]. Being in close contact with the dying relative until the end is highly important for family members with regard to the transition into a completely new life situation after bereavement. Finally death is "described [as] being ‘caught off guard’" by an informal caregiver [31].

#### Finding reorientation in a new life situation

After the death of the persons with palliative care needs, the bereaved family member or informal caregiver find themselves in a completely new life situation. The continuum − being in continuity or disruption – helps explain the reflections families have about life and death [21]. Being in disruption is characteristic for family members that have difficulties with accepting the situation and for whom it is challenging to find peace, while becoming overwhelmed by failings and losses. In contrast, being in continuity means being aware of the loss through death, while accepting the present and facing life [21], which is also described in the third subprocess of maintaining moments of hope [29]. Consequently, people will negotiate new priorities and activities with themselves after the death of a loved one [22], or family members consciously keep up traditions they once shared together [21]. Cherished memories of their common past are a source of comfort and confidence, and spiritual concepts like “immortality of the souls” provide support when facing a life without the partner, parent or child, thus easing the passage into adjusting to this new stage of life and preserving the will to persevere [21].

A conceptual model was derived based on the meta-synthesis of qualitative findings (Fig. 2). The analysis revealed three major themes characterizing the experiences of persons with palliative care needs and their family members with regard to transitions in the palliative context. The meta-synthesis indicated three main findings. First, persons with palliative care needs and their families adapt continuously to these changed life situations by maintaining the greatest degree of normality during transitions. Second, they use different strategies to become accustomed to the situation of finding themselves in changed roles. Third, they anticipate the future – persons with palliative care needs think about how relatives will continue without them, and relatives look ahead to their own future after their beloved family member has died.

## Discussion

The meta-synthesis resulted in a conceptual model in which the experiences of persons with palliative care needs and their families during transitions can be described. The main results of this meta-synthesis are as follows:

- Persons with palliative care needs and family members want to maintain normality in daily life while being confronted with new life situations due to transitions.
- They experience difficulties in dealing with their changed life situation and achieving balance.
- They encounter transitions with unconscious (passive) and conscious (active) adaptations.
- They have to deal with changing roles while anticipating the future.
- Supportive and distressing factors influence experiences and efforts made by persons with palliative care needs and their families to maintain normality in the face of death.

The central finding in this meta-synthesis *of maintaining normality in daily life* relates to adaptations by persons with palliative care needs and their families to unaccustomed and unprecedented life situations in the palliative context. Other studies also demonstrate that persons with palliative care needs and their families strive for normality in the palliative context [19, 33–36]. Families use self-management strategies to adapt to transitions [35]. Health care professionals can support family members and informal caregivers in their self-management strategies and therefore in maintaining normality [33, 35, 37]. Within this striving for normality, persons with palliative care needs and their families try to enjoy life through living in the moment [5, 34, 38]. Persons with palliative care needs and their families experience a narrowing lifeworld due to the restrictions imposed by the progressing illness. Their effort to maintain normality can be seen as a strategy to counteract their narrowing lifeworld [5]. Efforts to make use of resources strengthened and motivated both persons with palliative care needs and family members [38]. Hence, searching for normality in crisis situations has a central role. Health care professionals must be aware that maintaining normality can be established using different strategies and resources that they can also promote.

The meta-synthesis showed persons with palliative care needs and their families initially experience transitions unconsciously, until the situation cannot be dealt with anymore. This situation marks the transition and describes a crisis situation. Crisis can be understood as situation in which persons have to make decisions, change their behavior and find a new orientation [39]. In palliative situations at home, exacerbated symptoms such as acute worsening of the health status are perceived as a crisis [40]. Moreover, transitions are known to make concerned persons more vulnerable to health risks [1]. Experiencing this unprecedented situation, persons with palliative care needs and their families perceive the situation consciously and begin to adapt actively by elaborating new strategies to maintain “normality” and routines in daily life as best they can when faced with death. Therefore, transitions are an individual phenomenon that differ in number from one person to another. This result is contrasted by Penrod et al. [19], who describe certain key transitions for informal caregivers in the patient’s disease trajectory, from prediagnosis through to bereavement. Nevertheless, this meta-synthesis agrees with Penrod et al. by describing transitions as situations that challenge the established routine or “steady state” of the caregivers. While Penrod et al. only researched the caregivers perspective, this meta-synthesis can affirm the key process (“maintaining normality”) for persons with palliative care needs as well [19]. Consequently, transitions can be seen as unavoidable, natural processes in advanced illness [1, 19].

Persons with palliative care needs and their families all experience changing roles as the disease progresses. The meta-synthesis shows that persons with palliative care needs must deal with their role as persons with palliative care needs and their progressive inability to perform their former roles within the family. Meanwhile, family members are confronted with assuming new roles. The changing roles are a challenging experience for family members [5, 35]. Informal caregivers who succeed in their search for normality manage to conform to the complex demands of their role [19]. A positive decision to assume the caregivers role can be influenced by negative experiences with the residential home-care system [25] or because it is “self-evident and natural to care” [24]. Persons with palliative care needs and family members experience further alterations in well-being, habits and relationships as their roles change [38]. Assuming the caregivers role can have profound positive changes in the relationship between persons with palliative care needs and family members [36]. The relationship can also be characterized by conversations about the illness and enduring the uncertainty of the future together [5]. In contrast, some persons with palliative care needs do *not* want to talk about their illness and their imminent death. Often family members have a greater desire to talk about it, but see themselves confronted with barriers and sometimes feel isolated [34]. Family members in particular may have difficulty coping with the situation of accompanying a dying person and of facing the challenging demand to assume new roles on a constant basis. Contesting changes in roles costs effort. Family members have little freedom of choice when it comes to assuming a role, as persons with palliative care needs are no longer able to fulfil their roles. Consequently, a period of transition is also a time in which family members assume new roles in a constant process of negotiating the relationship with the persons with palliative care needs.

Also distressing and supporting factors could identified which either relieves or burdened the relatives during the transition phases. This can be explained with the concept of vulnerability. Vulnerability as a core concept was identified in a grounded theory study by Proot et al. (2003). In balancing between burden and capacity the authors found similar distressing and supporting aspects of relatives in terminal palliative care at home [41]. In our view, it is important that distressing and supportive factors in the transition phase are taken into account comprehensively.

Anticipating the future is important for persons with palliative care needs and for family members. While persons with palliative care needs try to imagine how life will continue without them, family members try to prepare for the death of a loved one and anticipate challenges after his or her death. Family members experience a new life situation in their role as a person who will lose a close family member in the near future. Within this new role, family members start to raise questions about their future alone or about economic aspects [35]. They think about how they can continue in the absence of their loved ones with palliative care needs. Awaiting imminent death is taxing for persons with palliative care needs and their family members [5]. Anticipating the future is combined with many feelings such as uncertainty, fear and loneliness [5, 42]. Further research will be necessary in order to address the issue of offering adequate support to family members and persons with palliative care needs.

This meta-synthesis enabled the derivation of a conceptual model about experiences of persons with palliative care needs and family members during transitions in the palliative context. A meta-synthesis helps to obtain a higher degree of abstraction. Nevertheless, the quality of the meta-synthesis may be limited by its dependence on the quality of the included studies. Furthermore, it was not possible to conduct the originally planned mixed-methods studies review due to the poor quality of the studies. Moreover, qualitative studies with moderate to insufficient quality have been integrated, as they held relevant content.

The findings help nurses to acquire understanding for persons with palliative care needs and their families in the palliative situation. As transitions result in a situation of change for the concerned persons, the knowledge about transitions can help nurses to recognize a transition. Consequently, they can assist those concerned in maintaining normality and designing strategies to adapt to transitions. Furthermore, they can support them in finding solutions, new strategies and in dealing with the situation, as they are familiar with the needs and experiences of concerned persons and their families in palliative situations.

## Conclusions

This study shows how persons with palliative care needs and their families experience transitions in a palliative context. Maintaining normality, experiencing changing roles and anticipating the future are central phenomena for these groups. The derived model of experiencing transitions in palliative situations shows a deviating view on transitions compared to the one promoted in traditional models. The results can help health care professionals understand persons with palliative care needs and their families. The health care professionals can use this understanding to offer targeted services, enabling the development of tailored education and counselling offers for persons with palliative care needs and family members/informal caregivers. The conceptual model assists in comprehending the experiences of persons with palliative care needs and their family members. Based on this model, health care professionals can identify available resources and support the family in their self-management strategies.

Further research is required to investigate the individual implications of bereavement after loved ones with palliative care needs have died. Given that a life-limiting illness has consequences for the family as a whole, another important topic will be to explore the changes within the lifeworld of persons with palliative care needs and family members as an integral unit.
